# NHE3 inhibitor tenapanor maintains intestinal barrier function, decreases visceral hypersensitivity, and attenuates TRPV1 signaling in colonic sensory neurons

**DOI:** 10.1152/ajpgi.00233.2023

**Published:** 2024-01-22

**Authors:** Andrew J. King, Lin Chang, Qian Li, Liansheng Liu, Yaohui Zhu, Pankaj J. Pasricha, Ji Wang, Matthew Siegel, Jeremy S. Caldwell, Susan Edelstein, David P. Rosenbaum, Kenji Kozuka

**Affiliations:** ^1^Ardelyx, Inc., Waltham, Massachusetts, United States; ^2^Geffen School of Medicine, University of California Los Angeles, Los Angeles, California, United States; ^3^Division of Gastroenterology, Department of Medicine, Johns Hopkins University School of Medicine, Baltimore, Maryland, United States

**Keywords:** dorsal root ganglia neurons, intestinal barrier permeability, tenapanor, TRPV1, visceral hypersensitivity

## Abstract

The pathogenesis of irritable bowel syndrome (IBS) is multifactorial, characterized in part by increased intestinal permeability, and visceral hypersensitivity. Increased permeability is associated with IBS severity and abdominal pain. Tenapanor is FDA-approved for the treatment of IBS with constipation (IBS-C) and has demonstrated improvements in bowel motility and a reduction in IBS-related pain; however, the mechanism by which tenapanor mediates these functions remains unclear. Here, the effects of tenapanor on colonic pain signaling and intestinal permeability were assessed through behavioral, electrophysiological, and cell culture experiments. Intestinal motility studies in rats and humans demonstrated that tenapanor increased luminal sodium and water retention and gastrointestinal transit versus placebo. A significantly reduced visceral motor reflex (VMR) to colonic distension was observed with tenapanor treatment versus vehicle in two rat models of visceral hypersensitivity (neonatal acetic acid sensitization and partial restraint stress; both *P* < 0.05), returning VMR responses to that of nonsensitized controls. Whole cell voltage patch-clamp recordings of retrogradely labeled colonic dorsal root ganglia (DRG) neurons from sensitized rats found that tenapanor significantly reduced DRG neuron hyperexcitability to capsaicin versus vehicle (*P* < 0.05), an effect not mediated by epithelial cell secretions. Tenapanor also attenuated increases in intestinal permeability in human colon monolayer cultures caused by incubation with proinflammatory cytokines (*P* < 0.001) or fecal supernatants from patients with IBS-C (*P* < 0.005). These results support a model in which tenapanor reduces IBS-related pain by strengthening the intestinal barrier, thereby decreasing permeability to macromolecules and antigens and reducing DRG-mediated pain signaling.

**NEW & NOTEWORTHY** A series of nonclinical experiments support the theory that tenapanor inhibits IBS-C-related pain by strengthening the intestinal barrier. Tenapanor treatment reduced visceral motor responses to nonsensitized levels in two rat models of hypersensitivity and reduced responses to capsaicin in sensitized colonic nociceptive dorsal root ganglia neurons. Intestinal permeability experiments in human colon monolayer cultures found that tenapanor attenuates increases in permeability induced by either inflammatory cytokines or fecal supernatants from patients with IBS-C.

## Graphical abstract

**Figure:**
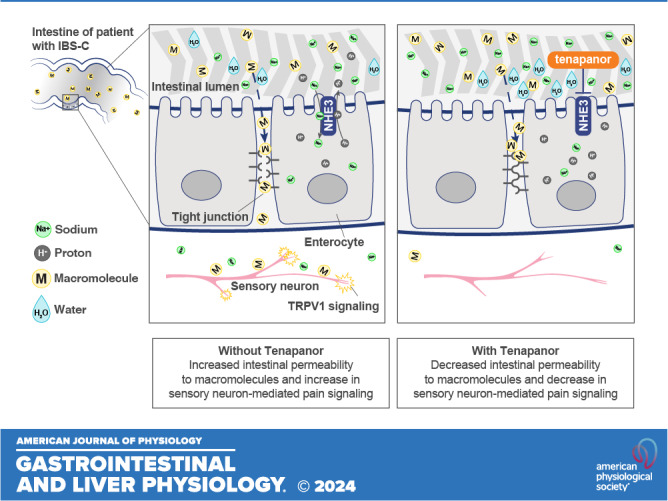

## INTRODUCTION

Irritable bowel syndrome (IBS) affects 4.7% of the adult population in the United States (1). Patients with IBS experience abdominal pain associated with bowel movements or changes in stool form or frequency (2). The pathogenesis of IBS is multifactorial, including motility disturbances, increased intestinal permeability, and visceral hypersensitivity, and alterations in gut microbiota (3–7). Increased permeability and mucosal barrier dysfunction in IBS may promote immune activation and visceral hypersensitivity (5, 8). IBS can be constipation-predominant (IBS-C), diarrhea-predominant (IBS-D), or associated with a mix of constipation and diarrhea (IBS-M) (2). Approximately 29% of patients with IBS in the United States have IBS-C (1).

Increased intestinal permeability is a common contributor to IBS pathogenesis. Exogenous markers of intestinal paracellular permeability that are poorly absorbed in a healthy gut [e.g., high molecular weight polyethylene glycol 3350 (PEG 3350) and dextrans] were increased in patients with IBS (9). Furthermore, increased paracellular permeability of these macromolecules correlated with IBS severity and abdominal pain (10, 11). In animal models, visceral hypersensitivity to colonic distension is dependent on increased paracellular permeability (12). Mucosal soluble mediators such as proinflammatory cytokines [e.g., tumor necrosing factor-α (TNF-α), interleukin 6 (IL-6)], and luminal factors in patients with IBS decrease transepithelial electrical resistance (TEER) of intestinal epithelial cells and increase paracellular permeability to macromolecules (13). Consistent with lower TEER, patients with IBS have decreased expression of tight junction proteins zonula occludens-1 (ZO-1) and occludin (14, 15).

Although over-the-counter and prescription treatments are available for IBS-C, only a minority of patients respond to these treatments (16). A survey of patients diagnosed with IBS-C found that, of those taking over-the-counter treatments, only 36% were satisfied or completely satisfied with treatment (16).

Although over-the-counter laxatives generally relieve constipation, they do not address the underlying pathophysiology or abdominal pain associated with IBS-C (17). There is a need for IBS-C treatments with different mechanisms of action. Several Food and Drug Administration (FDA)-approved treatments for IBS-C include guanylate cyclase-C agonists (linaclotide, plecanatide) and a chloride channel activator (lubiprostone) that increase chloride secretion in the gut (18–20).

Tenapanor is a first-in-class, minimally absorbed, small-molecule inhibitor of sodium/hydrogen exchanger isoform 3 (NHE3) with a novel mechanism of action (21). Tenapanor increases luminal sodium and water content and was approved by the FDA for the treatment of IBS-C in adults based on the results of the *phase 3* T3MPO-1 (NCT02621892) and T3MPO-2 (NCT02686138) studies (21–24). In *phase 3* clinical trials of tenapanor, 27%–37% of patients treated with tenapanor had increased bowel movements and a decrease in abdominal pain for 6 wk of 12 wk of treatment compared with 19%–24% of patients treated with placebo. The most commonly reported adverse event was diarrhea (22, 23). Preclinical and clinical studies of tenapanor demonstrate that the increased sodium and water retention in the intestinal lumen by inhibition of NHE3 facilitates softer stool consistency and accelerates transit (25–27). Studies with human intestinal epithelial cell monolayers showed that tenapanor modulates tight junctions and decreases the paracellular absorption of specific molecules (28); therefore, we hypothesized that tenapanor reduces abdominal pain in patients with IBS-C by restoring intestinal barrier function. Herein, we describe additional nonclinical studies that elucidate the mechanism of action of tenapanor for alleviating constipation and abdominal pain in IBS-C.

## MATERIALS AND METHODS

### Animal Models for Pharmacokinetic and Pharmacodynamic Experiments

Male Sprague-Dawley rats (aged ≈7 wk; Envigo, Livermore, CA) were individually housed in metabolic cages under a reverse light cycle (7:00 am–7:00 pm dark) and trained to meal feed for 6 days after 2 days of acclimation. On *day 7*, rats were dosed orally by gavage with vehicle (5 mL/kg) or tenapanor (0.15 mg/kg) immediately before their final meal. Separate cohorts (*n* = 6 each) were euthanized at 0.5, 1, 2, and 4 h postvehicle or tenapanor treatment. Before euthanasia, the gastrointestinal (GI) tract was surgically removed from each isoflurane-anesthetized animal with care to preserve luminal contents. The GI tract was sectioned into the proximal small intestine (pylorus to midpoint of the small intestine), distal small intestine (midpoint to the ileocecal junction), and cecum, and segments were ligated with clips to prevent leakage. Luminal contents of the sections were assessed for water content by calculating the difference in dry and wet weight and sodium concentration by ion chromatography. Luminal sodium content was calculated by multiplying luminal sodium concentration by luminal volume. Luminal concentration of tenapanor was measured by high-performance liquid chromatography using tandem mass spectrometry (HPLC-MS/MS; Agilent). Differences were assessed by the Student’s *t* test.

Sprague-Dawley rats (aged ≈7 wk; Charles River Laboratories, Hollister, CA) were doubly housed in microisolator cages and acclimated for ≥2 days before initiation of the study. Before treatment, rats were fasted overnight for 16 h, then treated with vehicle (deionized water) or tenapanor (10 mg/kg) at 5 mL/kg by oral gavage, followed by a 12% [weight per volume (wt/vol)] carmine red dye solution in deionized water by oral gavage. One set of animals (*n* = 6) was necropsied at 1 h posttreatment to determine the length of the small intestine from pylorus to ileocecal valve and the distance (centimeters) traveled by the red dye front. A second set of animals (*n* = 6) was necropsied at 6 h posttreatment to determine the length of the GI tract from pylorus to anus (centimeters) and the farthest length that the red dye was visible (centimeters). Transit through a given section of the GI tract was expressed as a percentage of the section that carmine red dye had traveled. The mean transit of the tenapanor-treated cohorts was compared with the mean of the vehicle cohorts using a Student’s *t* test where *P* value < 0.05 was considered significant.

### Animal Models of Hypersensitivity

Female Wistar rats (adult; Janvier SA, Le Genest St. Isle, France) were prepared for electromyography (EMG) by bilateral implantation of three pairs of nichrome wire electrodes in the striated muscles, 3 cm laterally from the midline. Cohorts of eight to nine rats were sensitized using partial restraint stress, as previously described (29), or nonsensitized (no partial restraint stress). Sensitized and nonsensitized rats were treated with tenapanor (3, 10, or 50 mg/kg orally) or vehicle (1 mL orally) 1 h before colorectal distension (CRD).

Sprague-Dawley rats (aged 10 days; Harlan Laboratories, Inc.) were sensitized using a 0.5% acetic acid colorectal infusion, as previously described (30), or nonsensitized using saline colorectal infusion. When sensitized and nonsensitized rats were aged ≥8 wk, a pair of electrodes was surgically implanted into their abdominal muscle tissue, followed by 1 wk of recovery. Cohorts of six to seven ≈ 10-wk-old rats were treated with tenapanor (1 or 10 mg/kg orally), MiraLAX (PEG 3350 1,000 mg/kg orally), or vehicle by mouth on the day of the experiment or with tenapanor (0.5 mg/kg orally), MiraLAX (1,000 mg/kg orally), or vehicle by mouth twice a day for 7 days before CRD.

### Assessment of Colorectal Distension

To induce CRD in female Wistar rats, a 4-cm long latex balloon fixed on a rigid catheter was inserted in the rectum at 1 cm from the anus, and a baseline measurement was performed at 0 mmHg. Isobaric distensions were performed from 0 mmHg to 60 mmHg at 15 mmHg increments by connecting the balloon to a computerized barostat. Each distension step lasted 5 min. Colonic pressure and balloon volume were continuously monitored on a potentiometric recorder (L6514, Linseis, Selb, Germany) with a paper speed of 1 cm/min. Electromyographic recordings were made from the implanted electrodes with an electromyography machine (Mini VIII; Alvar, Paris, France) using a short time constant (0.3 s) to remove low-frequency signals (<3 Hz). Abdominal contractions, as measured by EMG spike bursts, were quantified for a 5 min period at each distension pressure. Colorectal volumes were measured by a potentiometric recorder and defined as the maximum volume obtained at each stage of distention. Means were compared using an unpaired Student’s *t* test, with *P* value < 0.05 considered to be statistically significant.

The visceral motor reflex (VMR) response to CRD in Sprague-Dawley rats was conducted as previously described (30, 31). To summarize, a 5 cm balloon was inserted through the anus into the distal colon under isoflurane anesthesia, and the electrodes were connected for EMG recording. After 30 min equilibration, CRD was induced by rapidly inflating the balloon using a pressure transducer and sphygmomanometer to either 20, 40, 60, or 80 mmHg for 20 s with at least a 4 min interval between the application of pressures. VMR response to CRD was measured by EMG and normalized to baseline EMG values measured 20 s before CRD. The EMG signal was amplified with a low-noise alternating current (AC) differential amplifier, filtered at 10–3,000 Hz, digitized, and integrated using the CED (Cambridge Electronic Design) 1401/SPIKE2 program (CED, Cambridge, UK). The raw EMG data were rectified and quantified by calculating the area under the curve (AUC). Then, the VMR responses to CRD were calculated by subtracting baseline AUC from the AUC of CRD. The data were presented as means ± SE. Groups were compared statistically by two-way analysis of variance (ANOVA) using SigmaPlot (Systat Software, Inc., San Jose, CA).

### Dorsal Root Ganglia Neuron Patch-Clamp Recordings

DRG sensory neurons that project to the colon were labeled in vivo by injecting a red fluorescent retrograde dye, FAST DiI (1,1’-dilinoleyl-3,3,3',3'-tetramethylindocarbocyanine perchlorate, 10 mg/dL in methanol) into the distal colon wall (2 µL/site, 10 sites/rat). The DRGs (T12-L1 and L4-S1) were dissected from euthanized sensitized and nonsensitized Sprague-Dawley rats (see *Animal models of hypersensitivity*) (30) that were treated with tenapanor (0.5 mg/kg orally) or vehicle (3 mL/kg) twice a day for 7 days beginning 1 wk after FAST DiI injection. Three hours after euthanasia, neuronal excitability and capsaicin response of DiI-labeled DRGs were measured using whole cell voltage patch-clamp recordings as previously described (32).

To test whether the effects of tenapanor are mediated by secretions from enteric epithelial cells, dissociated DRG neurons from adult nonsensitized Sprague-Dawley rats were incubated for 24 or 48 h in a conditioned medium harvested from the basolateral chamber of human colonic enteroid monolayers pretreated on the apical side with tenapanor (1 μM) or vehicle. Whole cell voltage patch-clamp recordings were performed as described previously (32).

Chinese hamster ovary (CHO) cells with recombinant human TRPV1 were cultured and treated with tenapanor at concentrations ranging from 9.0 × 10^−11^ M to 3.0 × 10^−5^ M before exposure to TRPV1 agonist (30 nM capsaicin) at room temperature in a calcium flux assay using capsazepine as the positive control inhibitor (33). Cellular antagonist effect was calculated as a percentage inhibition of capsaicin response.

### Cell Models of Permeability

Primary human transverse colon monolayer cultures were established using cells derived from three-dimensional human intestinal organoids, which were grown from human colon biopsies, as described previously (34). For example, human colon monolayer cultures recapitulate in vivo cellular composition and function, including the formation of tight junctions and TEER of ∼500 Ω·cm^2^ after 5–6 days in culture (35). Cell cultures in 24-well Transwell plates (Corning) were treated for 16–48 h with recombinant TNF-α (1–100 ng/mL; PeproTech), recombinant IL-6 (1–100 ng/mL; PeproTech), or fecal supernatants from healthy controls or patients with IBS-C (diluted 1:4), in the presence of tenapanor (1 μM) or DMSO control. The final concentration of DMSO in the media [advanced Dulbecco’s modified Eagle medium/Ham’s F-12 (DMEM/F12; Thermo Fisher)] was 0.1%. TNF-α and IL-6 were added to the apical and basolateral sides of the cell monolayer to simulate mild inflammation, which can occur in patients with IBS (36). The fecal supernatant was added only to the apical (luminal) side. Cells were incubated in a 37°C, 5% CO_2_ incubator. Fecal supernatants were prepared by dissolving fecal samples at a concentration of 0.3 g/mL in oxygenated Krebs–Ringer buffer and homogenizing on ice with a Polytron homogenizer (30 s, 26,000 rpm). After centrifugation (10,000 *g*, 10 min, 4°C), the supernatants were recovered, and coarse particles were separated by filtration using a 100 μm-sized filter. The barrier integrity of the monolayer was assessed by measuring TEER, and apical-basolateral flux of the paracellular marker, 4 kDa fluorescein isothiocyanate-dextran (FITC-dextran). FITC-dextran (Sigma Aldrich) was prepared in media at a final concentration of 1 mg/mL and added to the apical side of the monolayer. Media samples from the basolateral side and FITC-dextran calibration standards (0.1–1,000 ng/mL) were placed in a 96-well plate and fluorescence was measured with a SpectraMax M2 plate reader (Molecular Devices) (excitation: 485 nm, emission: 530 nm). TEER was measured at room temperature using a Millicell Electrical Resistance System (ERS)-2 V-ohmmeter (MilliporeSigma) immediately after the basolateral samples were collected from the FITC-dextran assay. Data are presented as means ± SD. *P* values were calculated using a two-way, repeated-measures ANOVA for the inflammatory cytokine experiments. In the study of tenapanor’s effect on FITC-dextran permeability with fecal supernatants, data are presented as a mean of three values for each fecal supernatant from nine patients with IBS-C and 10 healthy controls. *P* values were calculated by comparing DMSO with 1 µL tenapanor using a Wilcoxon-matched pair signed rank test.

### *Phase 1* Study Design and Participants

Pharmacokinetics and pharmacodynamics of multiple-ascending doses of tenapanor were evaluated in 40 healthy male and female volunteers [aged: 19–65 yr; body mass index (BMI): 18–29.9 kg/m^2^] during a single-center, randomized, double-blind, placebo-controlled study. The study design has been described previously (27). To summarize, participants were randomized 4:1 to receive tenapanor (3 mg, 10 mg, 30 mg, or 100 mg) or placebo once a day (daily) for 7 days. The median time to the first bowel movement was measured from the time of the morning treatment dose to the first bowel movement experienced by the study participant, using a product-limit analysis.

### Ethical Conduct

Animal welfare was maintained in accordance with the *Guide for the Care and Use of Laboratory Animals* (National Research Council 2011). All animal protocols were approved by a local Institutional Animal Care and Use Committee. The protocol for the *phase 1* pharmacokinetics and pharmacodynamics study in humans was approved by a local institutional review board (Integreview Ethical Review Board, Austin, TX) and was registered on ClinicalTrials.gov (NCT02819687) (27). Volunteers provided written informed consent before enrolling.

### Statistical Methods

Data are summarized using descriptive statistics as mean (SE) unless otherwise stated. A two-tailed, unpaired Student’s *t* test or two-tailed ANOVA was used to assess the statistical significance of differences between groups, as stated above.

## RESULTS

### Intestinal Pharmacokinetics and Pharmacodynamics of Tenapanor and Effect on Constipation

**Figure 1.**
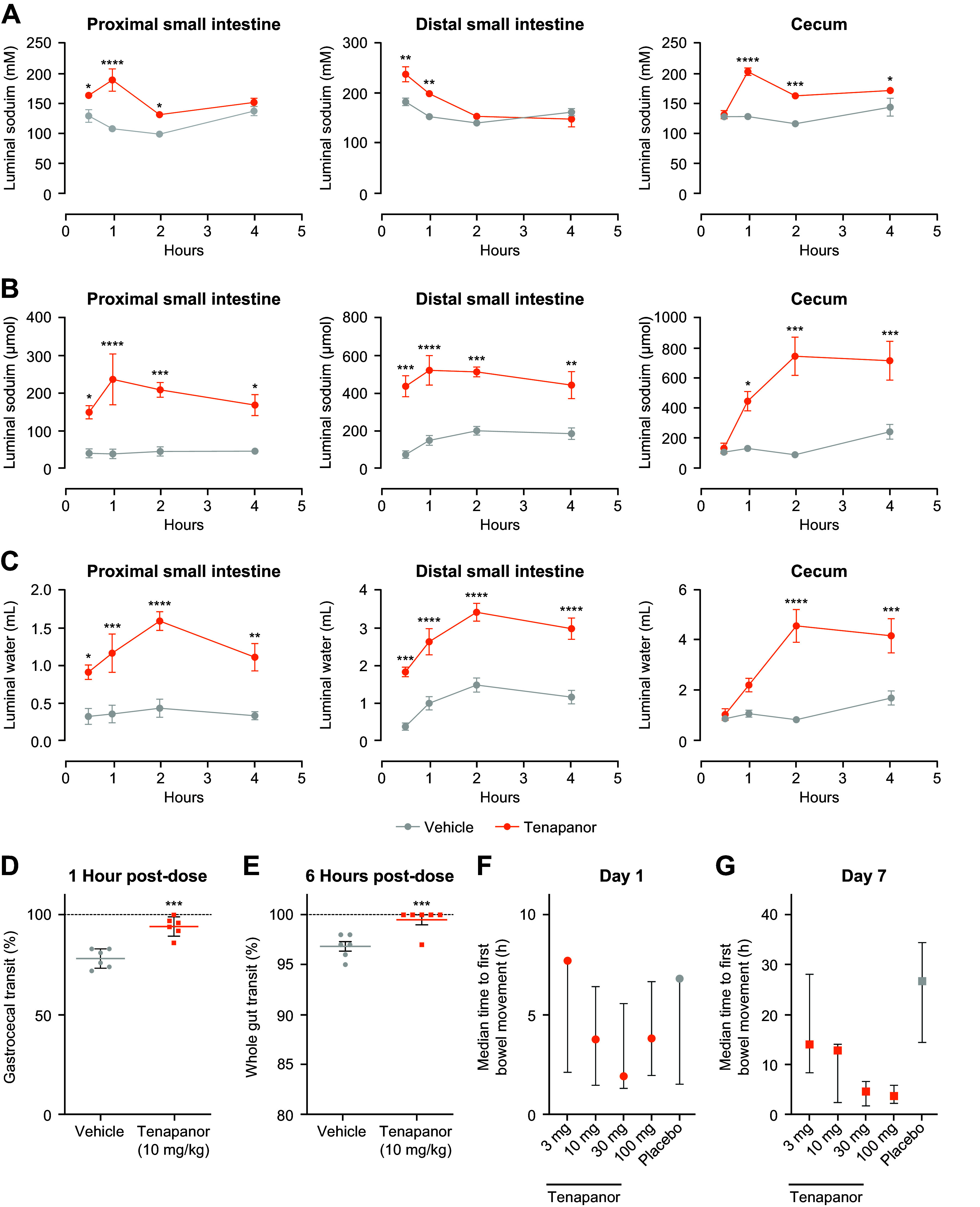
Effects of oral tenapanor treatment (0.15 mg/kg) on luminal content and gastrointestinal transit. *A*–*C*: pharmacodynamics of tenapanor in the proximal small intestine, distal small intestine, and cecum (*n* = 6 rats/cohort). *P < 0.05 vs. vehicle, **P < 0.01 vs. vehicle, ***P < 0.001 vs. vehicle, ****P < 0.0001 vs. vehicle (two-way ANOVA, with post hoc Sidak’s multiple comparisons). *D* and *E*: transit of carmine red through the gastrointestinal tract of rats at 1 h and 6 h posttreatment with tenapanor or vehicle (*n* = 6 rats/cohort). Statistics are a two-tailed, unpaired *t* test, ***P < 0.01. *F* and *G*: time to first bowel movement in humans was measured from the time of the first morning dose of tenapanor (3 mg, 10 mg, 30 mg, and 100 mg) or placebo. Data are presented as medians. Error bars represent the 25th and 75th percentiles. A post hoc analysis using a log-rank test found a significantly lower median time to first bowel movement for all tenapanor groups vs. placebo (overall treatment difference, *P* < 0.05). ANOVA, analysis of variance.

Tenapanor is minimally absorbed by design, which results in higher luminal concentrations of the drug (data not shown); this led to increases in luminal sodium and water retention in rats after a single dose (Fig. 1, *A*–*C*). Sodium and water were significantly increased in the lumen of the proximal and distal small intestine as early as 30 min after treatment, with tenapanor compared with vehicle control (proximal, *P* < 0.05; distal, *P* < 0.01). In the cecum, sodium was significantly increased with tenapanor versus vehicle as early as 1 h after treatment, followed by a significant increase of water after tenapanor treatment versus vehicle at 2 h posttreatment (*P* < 0.0001 for both).

Treatment with tenapanor increased luminal fluid, which led to faster GI transit of a nonabsorbed dye/marker in rats (Fig. 1, *D*–*E*). In rats treated with tenapanor, carmine red dye migrated significantly further after 1 h compared with vehicle (94.2 ± 2.0% vs. 78.2 ± 2.0%, respectively; *P* < 0.01; Fig. 1*D*). After 6 h, the dye front in the vehicle control group had traversed a mean 96.8% of gastroanal distance; however, none of the six animals had dye in their feces. In contrast, the dye front in tenapanor-treated animals traversed a mean 99.5% of the gastroanal distance, and five of six animals had dye in their feces. (Fig. 1*E*).

In humans, faster time to first bowel movement was seen with tenapanor compared with placebo (Fig. 1*F*). Healthy volunteers treated with higher doses of tenapanor (30 mg daily and 100 mg daily) had a lower median time to first bowel movements than participants treated with lower doses of tenapanor (3 mg daily and 10 mg daily) or placebo (Fig. 1*F*).

### Effects of Tenapanor on Visceral Hypersensitivity

Two rat models were used to study the effects of tenapanor on visceral hyperalgesia. The first model was a stress model that induces visceral hyperalgesia from the brain to the gut (top to bottom), whereas the second model was a neonatal colorectal irritation model (bottom to top).

**Figure 2.**
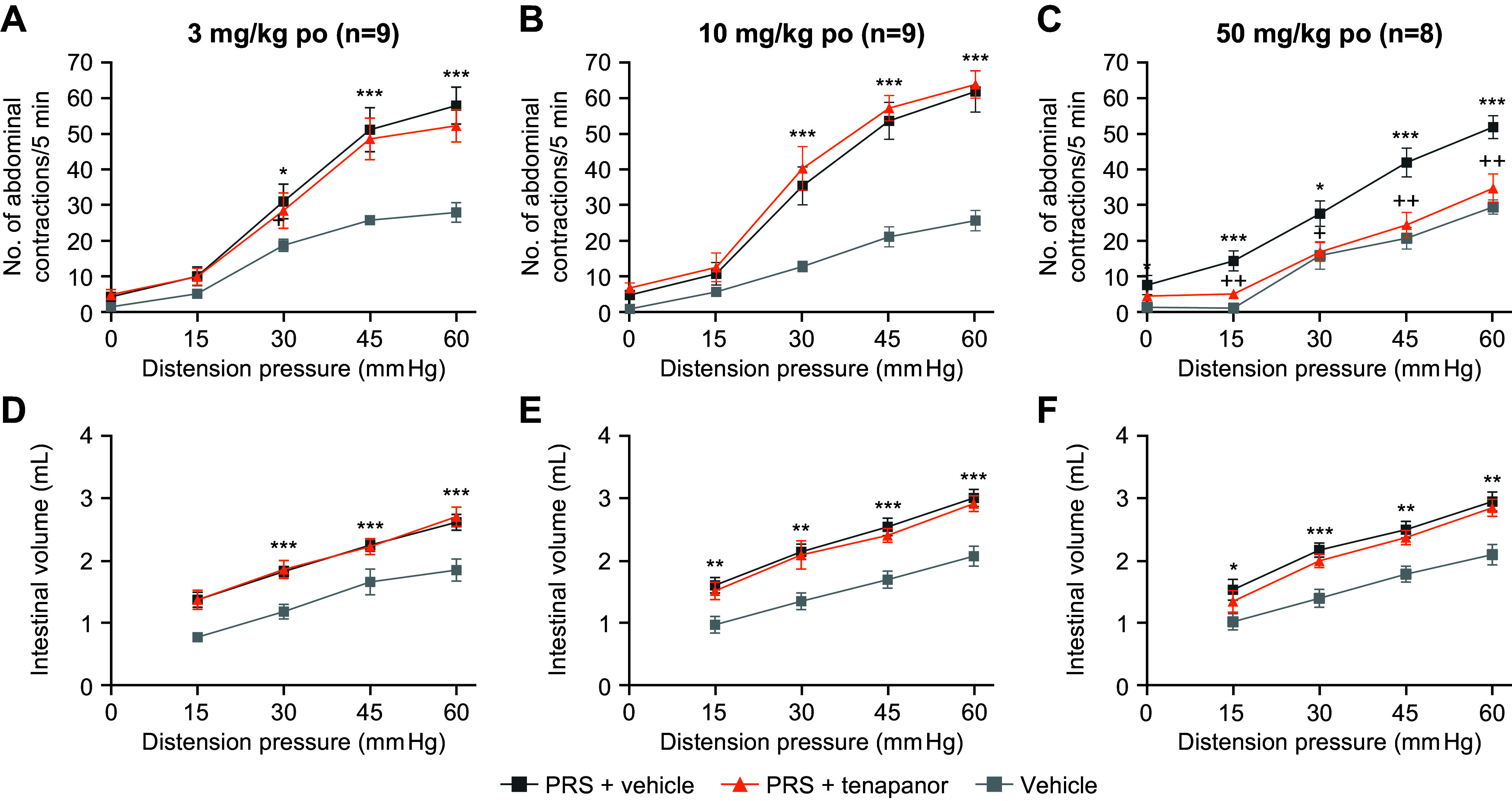
Assessment of the VMR in response to CRD in a PRS model in female Wistar rats. Tenapanor or vehicle were administered orally 1 h before CRD. Tenapanor treatment at 3 or 10 mg/kg produced similar VMR to CRD as rats treated with vehicle (*A* and *B*; *n* = 9 rats/cohort), whereas tenapanor (50 mg/kg) induced a significantly smaller VMR than vehicle, similar to rats who did not undergo PRS (*C*; *n* = 8 rats/cohort). Intestinal volume was consistent between stress models (*D*−*F*). Data are presented as means ± SE. Significantly different from vehicle: *P < 0.05, **P < 0.01, ***P < 0.001. Significantly different from PRS + vehicle: +*P* < 0.05, ++*P* < 0.01. CRD, colorectal distension; po, by mouth; PRS, partial restraint stress; VMR, visceral motor reflex.

A dose-dependent reduction in visceral hypersensitivity to CRD was demonstrated in a partial restraint stress model in female Wistar rats treated with tenapanor (Fig. 2, *A*–*C*). In the absence of partial restraint stress, treatment with tenapanor produced no effect on colorectal sensitivity or intestinal volumes induced by CRD compared with vehicle (Fig. 2, *D*–*F*). After partial restraint stress, tenapanor 50 mg/kg abolished stress-induced colorectal hypersensitivity to CRD (*P* < 0.05 at all pressures, Fig. 2*C*).

**Figure 3.**
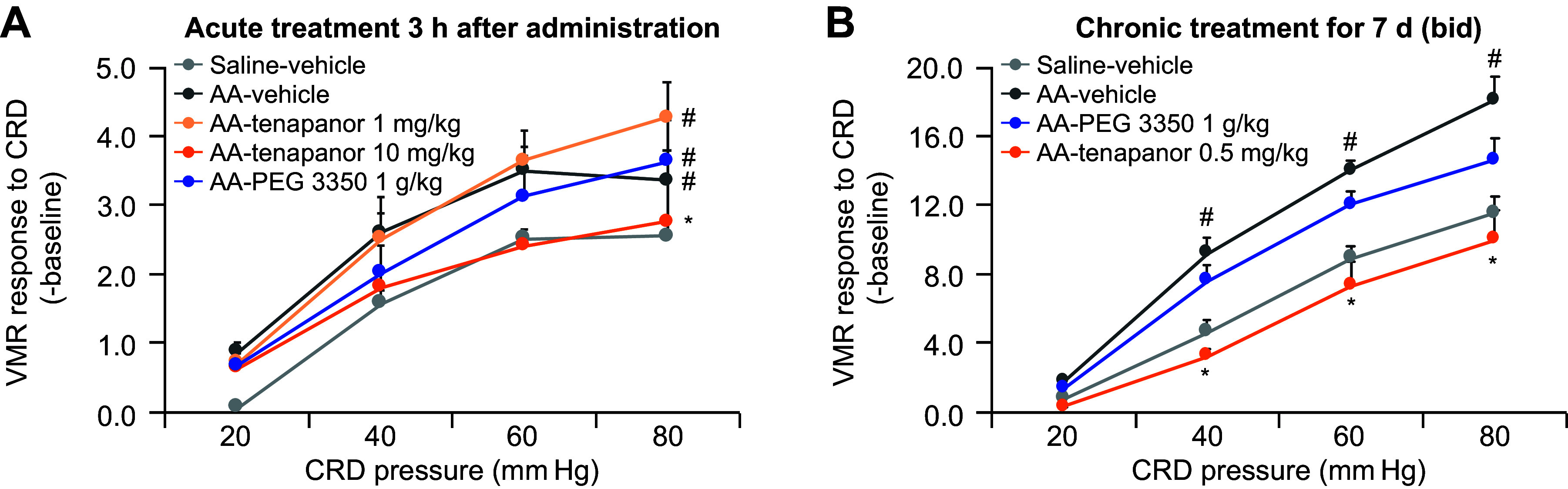
Effects of acute (*A*) and chronic (*B*) treatment of a Sprague-Dawley rat model of IBS induced by neonatal acetic acid sensitization. Rats were treated orally with tenapanor (1 or 10 mg/kg), MiraLAX (1,000 mg/kg), or vehicle (*n* = 6 or 7/cohort). Acute and chronic tenapanor treatment decreased pain responses as measured by a decrease in VMR to CRD vs. sensitized, vehicle-treated rats. Acute and chronic MiraLAX treatment of sensitized rats still resulted in increased pain responses vs. nonsensitized, vehicle-treated rats. Data are presented as means ± SE. Two-way ANOVA showed *P* < 0.05 for the main effects of treatment and pressure. #Significant difference from saline-vehicle group with overall comparison (*A*) or at same pressure (*B*); *significant difference from AA-vehicle with overall comparison (*A*) or at same pressure (*B*). AA, acetic acid; ANOVA, analysis of variance; bid, twice daily; CRD, colorectal distension; IBS, irritable bowel syndrome; VMR, visceral motor reflex.

Tenapanor prevented visceral hypersensitivity response and normalized colonic sensory neuronal excitability in a rat model of IBS following single and repeated oral administration (Fig. 3). Neonatal acetic acid treatment of male Sprague-Dawley rats significantly increased pain sensitivity (data not shown). Single treatment with tenapanor (10 mg/kg orally) significantly reduced neonatal acetic acid-induced hypersensitivity as measured by VMR response to CRD compared with vehicle (main effect of treatment, *P* < 0.05). On the other hand, a single treatment with 1 mg/kg tenapanor or 1,000 mg/kg PEG had no effect on hypersensitivity compared with vehicle (Fig. 3*A*). When neonatal acetic acid-sensitized rats were treated repeatedly with tenapanor 0.5 mg/kg, PEG, or vehicle twice a day for 7 days, tenapanor treatment significantly reduced VMR response to CRD compared with a vehicle to nonsensitized rat levels (main effect of treatment, *P* < 0.05); PEG had no significant effect on hypersensitivity (Fig. 3*B*).

### Effects of Tenapanor on Hypersensitivity Are Mediated by Hyperactive Sensory DRG Neurons

**Figure 4.**
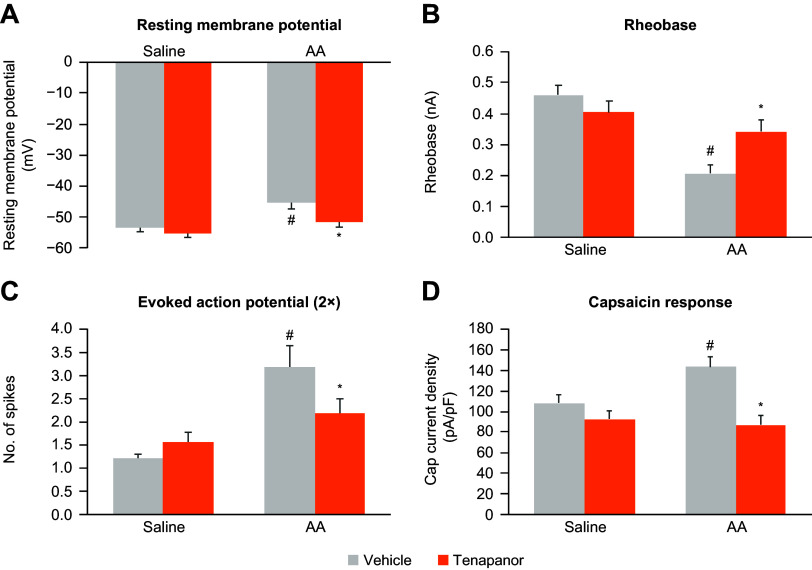
Effects of twice-daily, 7-day treatment with oral tenapanor (0.5 mg/kg) on the excitability of colon-projecting neurons in the DRG of acetic acid-sensitized rats (*n* = 23–35 cells). The excitability of DiI-labeled DRG neurons were examined by resting membrane potential (*A*), rheobase (*B*), 2x-rheobase-evoked action potential (*C*), and response to capsaicin (*D*). The data are presented as means ± SE. In all four measurements, two-way ANOVA revealed significant differences (*P* < 0.05) in both main effects of model and treatment. #Significant difference from saline-vehicle group; *significant difference from AA-vehicle group. AA, acetic acid; ANOVA, analysis of variance; DRG, dorsal root ganglia.

Tenapanor reduced the excitability of colonic innervated DRGs in neonatal acetic acid-sensitized rats in vivo (Fig. 4, *A*–*C*). We first tested whether the effects of tenapanor on hypersensitivity were mediated by a reduction in DRG activity. Retrograde dye (DiI) was injected into the colon and traveled to DRGs that projected to the colon. DRGs from acetic acid-sensitized rats had increased excitability compared with nonsensitized rats as demonstrated by an increase in the resting membrane potential, a reduction in rheobase, and an increase in the number of evoked action potential spikes (main effect of model, *P* < 0.05 for all measures; Fig. 4, *A*–*C*). Treatment of acetic acid-sensitized rats with tenapanor twice a day for 7 days significantly reduced the excitability of DRGs compared with vehicle (main effect of treatment, *P* < 0.05 for all measures), whereas, tenapanor treatment had no effect on DRGs from nonsensitized rats (Fig. 4, *A*–*C*).

In single-cell electrophysiology studies of rat colonic DRG sensory neurons, response to capsaicin was significantly increased in DRG neurons from acetic acid-sensitized rats (main effect of model, *P* < 0.05; Fig. 4*D*), suggesting increased TRPV1 signaling, which may mediate the observed increase in sensitivity to CRD (Fig. 3). Tenapanor treatment significantly reduced the hyperexcitability of DRG neurons compared with vehicle treatment in sensitized rats (main effect of treatment, *P* < 0.05) but had no effect in nonsensitized animals, suggesting that tenapanor normalized colonic sensory neuronal excitability and TRPV1 currents (Fig. 4*D*).

**Figure 5.**
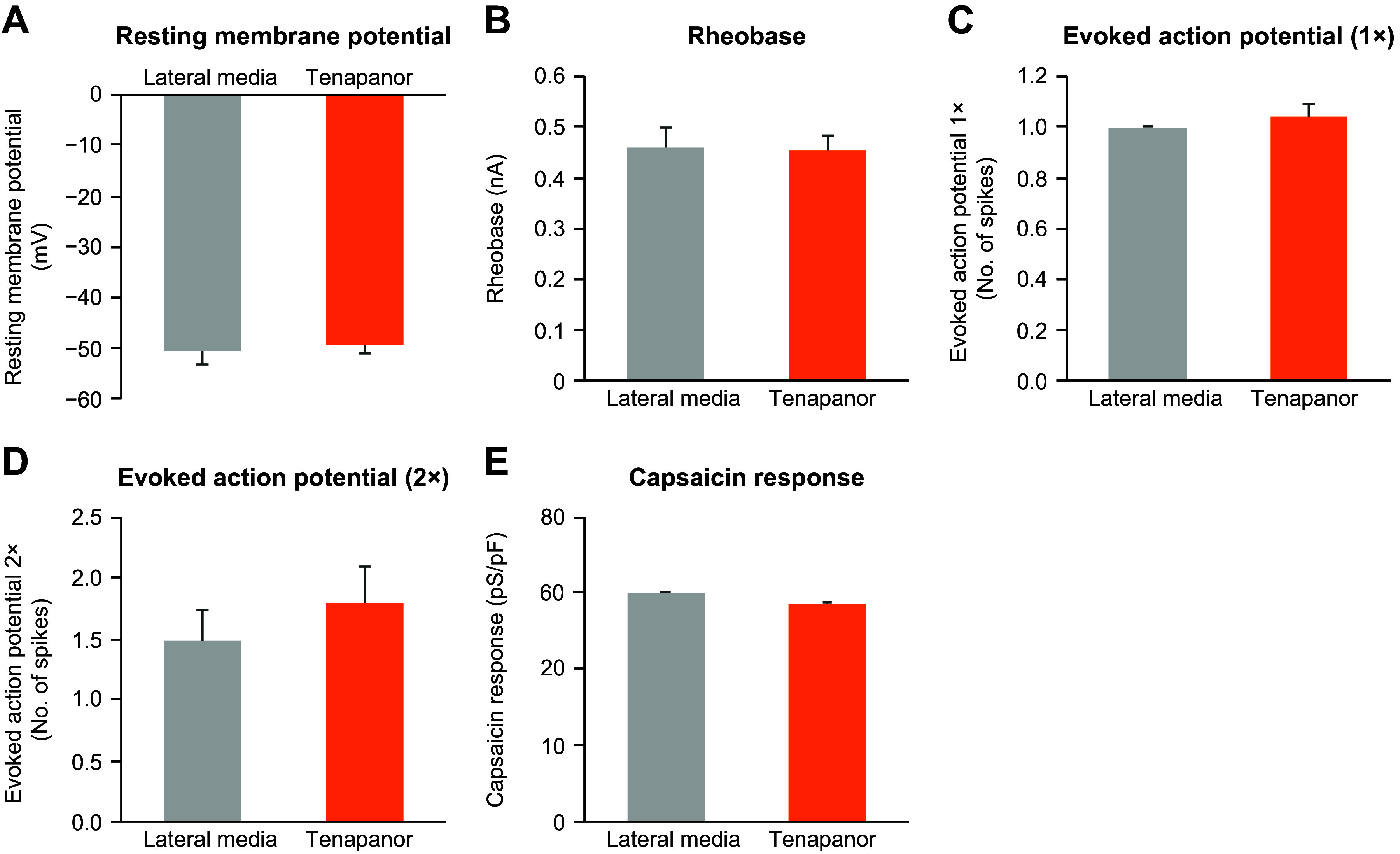
Effects of basolateral-conditioned media from human colon monolayers treated with or without tenapanor (1 μM) on rat DRG excitability (*A*−*D*) and capsaicin response (*E*). Rat DRG neurons were incubated with conditioned media for 24 h, and patch-clamp recording was then conducted (*n* = 20–24 cells). The data are presented as means ± SE. No significant difference between the media from treatment with tenapanor and control was detected by Student’s *t* test. DRG, dorsal root ganglia.

To test whether the effects of tenapanor are mediated by tenapanor-induced secretions from epithelial cells, we incubated DRG neurons with conditional media from a tenapanor-treated colon monolayer and then investigated DRG neuronal activity. No difference was seen between DRGs incubated with tenapanor-treated and vehicle-treated conditioned basolateral media in terms of neuronal excitability and response to capsaicin, suggesting that the secretome from epithelial cells is not responsible for mediating the reduction in neuronal excitability associated with tenapanor (Fig. 5).

Finally, we tested whether tenapanor directly inhibits TRPV1 by testing its effects on CHO cells that express human TRPV1. Tenapanor at concentrations ranging from 3.0 × 10^−5^ M to 9.0 × 10^−11^ M did not inhibit human TRPV1 response to capsaicin (data not shown), suggesting that although tenapanor affects TRPV1 signaling in DRG neurons, it does not directly inhibit the TRPV1 channel.

### Impact of Tenapanor on Intestinal Permeability

**Figure 6.**
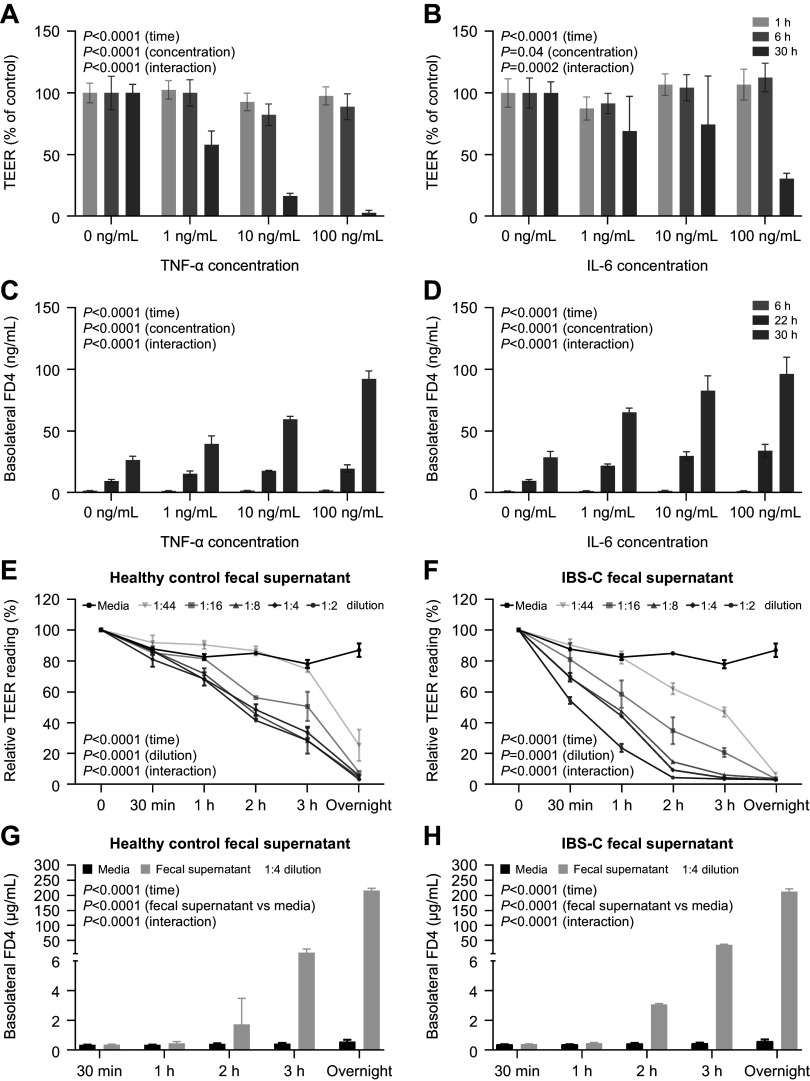
Treatment of human colon monolayers with cytokines TNF-α and IL-6 resulted in a dose- and time-dependent decrease in TEER (*A* and *B*) and increase in FITC-dextran permeability (*C* and *D*). Treatment of human colon monolayers with fecal supernatants from healthy controls (*n* = 10) and patients with IBS-C (*n* = 9) results in dose- and time-dependent reductions in TEER (*E* and *F*) and increases in FITC-dextran permeability (*G* and *H*). Data are presented as mean ± SD. *P* values were calculated using a two-way, repeated-measures ANOVA. ANOVA, analysis of variance; FD4, fluorescein isothiocyanate-dextran; FITC, fluorescein isothiocynate; IBS-C, irritable bowel syndrome with constipation; IL-6, interleukin 6; TEER, transepithelial electrical resistance; TNF-α, tumor necrosis factor α.

**Figure 7.**
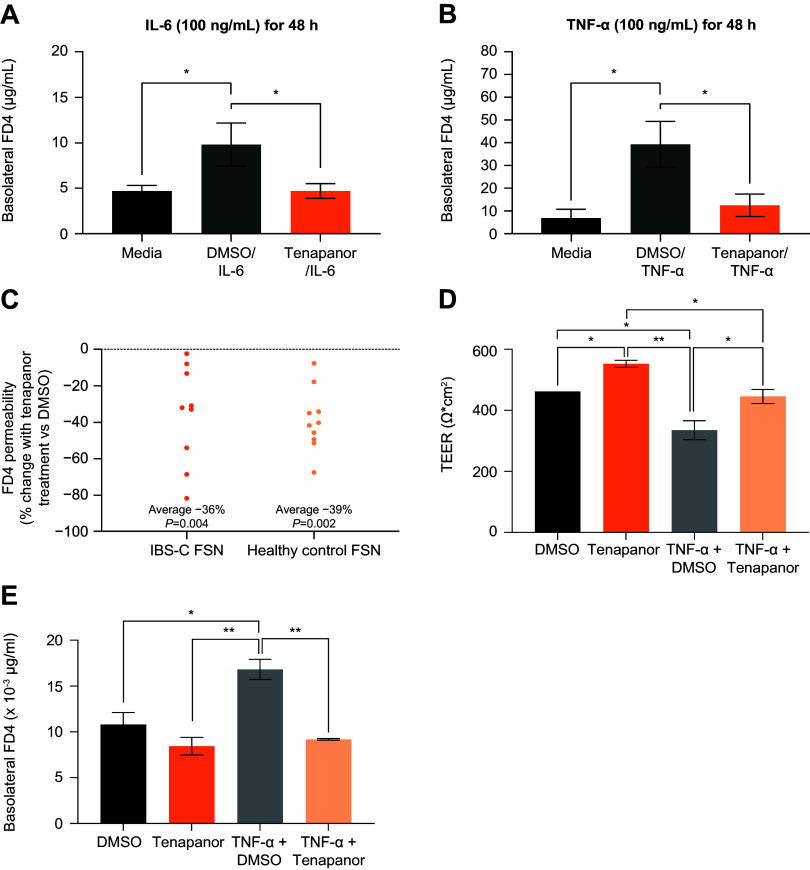
FITC-dextran permeability of human colon monolayers treated with or without tenapanor (1 μM) and IL-6 (*A*), TNF-α (*B*), or fecal supernatant (*C*) from healthy controls (*n* = 10) or patients with IBS-C (*n* = 9). Monolayers were incubated with fecal supernatant for 16 h. Inflammatory cytokine results are presented as means ± SD. *P < 0.001, two-tailed Student’s *t* test. Fecal supernatant results are presented as means of three values each for fecal supernatants from 9 patients with IBS-C and 10 healthy controls. *P* values were calculated using a Wilcoxon-matched pairs signed rank test. *D* and *E*: effects of DMSO (control), tenapanor (1 µM), TNF-α plus DMSO, or TNF-α plus tenapanor on TEER (*D*) and FITC-dextran permeability (*E*) of human colon monolayers (*n* = 2/group). Monolayers were incubated with the treatments for 23 h. Data are presented as means ± SD. *P < 0.05, **P < 0.01, One-way ANOVA with Tukey’s post hoc test. DMSO, dimethyl sulfoxide; FD4, fluorescein isothiocyanate-dextran; FITC, fluorescein isothiocynate; FSN, fecal supernatant; IBS-C, irritable bowel syndrome with constipation; IL-6, interleukin 6; TEER, transepithelial electrical resistance; TNF-α, tumor necrosis factor-α.

As the effects of tenapanor on DRG hyperexcitability could be downstream of changes to intestinal permeability (10), we next probed the influence of tenapanor on intestinal barrier function under IBS-like conditions using primary human colon monolayer cultures treated with the proinflammatory cytokines TNF-α and IL-6, or fecal supernatants from patients with IBS-C and healthy controls. We initially evaluated various concentrations and incubation times to determine optimal treatment parameters. Treatment with TNF-α and IL-6 resulted in time- and concentration-dependent reductions in TEER and an increase in permeability to macromolecule FITC-dextran, with the greatest effects observed at concentrations of 100 ng/mL over 30 h of incubation (Fig. 6, *A*–*D*). Similarly, treatment of human colon monolayer cultures with fecal supernatants from patients with IBS-C and healthy controls both resulted in time- and concentration-dependent decreases in TEER and a time-dependent increase in FITC-dextran permeability over 24 h (Fig. 6, *E*–*H*). Tenapanor treatment significantly attenuated the increase in permeability of human colon monolayer cultures to FITC-dextran caused by treatment with TNF-α (100 ng/mL for 48 h, *P* < 0.001), IL-6 (100 ng/mL for 48 h, *P* < 0.001), and fecal supernatants (1:4 dilution for 16 h, *P* < 0.005, Fig. 7, *A*–*C*).

The integrity of the human colon monolayers treated with TNF-α (100 ng/mL for 23 h) was confirmed by TEER measurements, which were comparable with those previously reported for untreated human colon monolayers (≥300 Ω×cm^2^, Fig. 7*D*) (36). Furthermore, tenapanor (1 µM) treatment significantly increased TEER of the TNF-α-treated human colon monolayers to levels observed in untreated control (DMSO) monolayers (429–462 Ω × cm^2^, Fig. 7*D*). Similarly, in the same TNF-α-treated human colon monolayers, tenapanor treatment decreased FITC-dextran permeability to that observed in untreated control (DMSO) monolayers (Fig. 7*E*). Tenapanor treatment of control human colon monolayers increased TEER by ∼20% (Fig. 7*D*), similar to the increase in TEER reported in human duodenum monolayers treated with tenapanor (28). However, tenapanor did not have any significant effect on the FITC-dextran permeability of control human colon monolayers (Fig. 7*E*), which was expected as FITC-dextran is a high-molecular-weight marker with inherently low paracellular permeability in untreated intestinal epithelial cells.

## DISCUSSION

**Figure 8.**
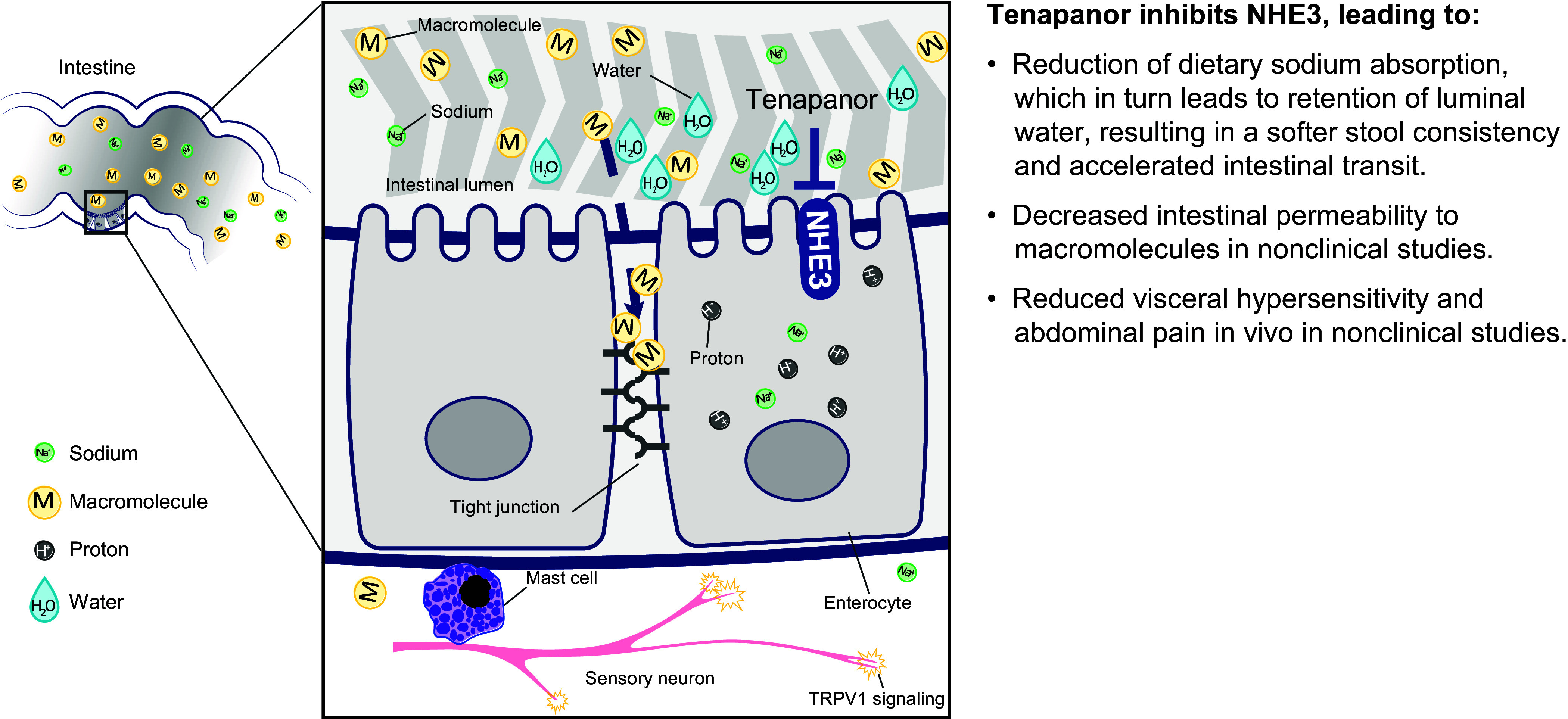
Schematic of proposed mechanism of action of tenapanor. Tenapanor inhibits NHE3, producing multiple downstream effects in the digestive tract. Reduction in dietary sodium intake leads to increased luminal water retention, allowing for faster gastrointestinal transit. Tenapanor also decreases intestinal permeability to macromolecules and antigens and reduces abdominal pain signaling. NHE3, sodium/hydrogen exchanger isoform 3; TRPV1, transient receptor potential cation channel subfamily V member 1.

Tenapanor inhibits the intestinal NHE3 transporter and reduces dietary sodium absorption, resulting in therapeutic effects for both GI motility and abdominal pain (Fig. 8). Tenapanor, which is minimally systemic, has demonstrated efficacy and safety in improving chronic constipation experienced by patients with IBS-C in *phase 2* and *phase 3* clinical studies (21, 24). In both animal and human studies, tenapanor reduced urine sodium and increased fecal sodium content (25, 27), which was associated with increased water retention in the gut. The clinical relevance of this mechanism of action was seen in the *phase 2b* and *phase 3* IBS-C studies, where tenapanor treatment relieved constipation by significantly increasing complete spontaneous bowel movement frequency (22, 23).

Tenapanor also reduces abdominal pain in patients with IBS-C (22, 23). Chronic abdominal pain is a hallmark of IBS (2), which may relate to increased visceral hypersensitivity (5). Treatments for IBS-C that treat constipation do not typically affect associated pain despite a loosening of stools, suggesting that pain associated with IBS-C arises from a distinct mechanism other than distention induced by constipation (37). Here, PEG treatment resulted in loosened stools without affecting the VMR response to CRD. In contrast, tenapanor treatment loosened stools while also reducing the VMR response to CRD. These results are aligned with prior studies of tenapanor. In the two *phase 3* T3MPO-1 and T3MPO-2 studies, reductions in abdominal pain with tenapanor compared with placebo were observed within the first week of treatment, along with improvements in global symptoms of IBS (22, 23). Here, tenapanor treatment in two different models of visceral hypersensitivity decreased sensitivity to CRD. In contrast, treatment with the over-the-counter laxative MiraLAX had no significant effect, aligned with patient reports of dissatisfaction and lack of efficacy on abdominal pain associated with laxatives for IBS (38). These results suggest that the reduction in abdominal pain observed in clinical trials of tenapanor treatment may result from mechanistic effects on pain reception rather than changes in GI motility.

TRPV1 is a well-known pain receptor that responds to a variety of noxious stimuli (30). Akbar and colleagues found that TRPV1 expression on nerve fibers was 3.5 times higher in the colon tissue of patients with IBS versus healthy controls and was correlated with the abdominal pain score (39). Tenapanor treatment did not directly inhibit recombinant human TRPV1 activation in CHO cells and is unlikely to act directly on other nociceptive neuron receptors in vivo based on prior pharmacokinetics data. After an oral dose of ^14^C-radiolabeled tenapanor to rats, nearly all the dosed radioactivity was recovered in feces as unchanged ^14^C-labeled tenapanor, with very low and transient levels of radioactivity in the colon tissue (25). Therefore, direct exposure to tenapanor of the submucosal and myenteric plexus, where DRG neurons reside, is expected to be limited in vivo.

As tenapanor is minimally absorbed (25), direct action on TRPV1 is unlikely, and tenapanor may instead indirectly decrease TRPV1-mediated pain signaling. Patients with IBS and sensitized animals display increased intestinal permeability associated with abdominal pain (5, 7, 10–12). This increase in permeability could promote TRPV1-mediated pain signaling through the activation of an inflammatory immune response (3). Increased permeability could enable translocation of bacteria and associated antigens (e.g., lipopolysaccharide), which are elevated in the feces and serum of patients with IBS (40, 41). Increases in fecal and serum lipopolysaccharide are also observed in rats fed a high fermentable oligosaccharides, disaccharides, monosaccharides, and polyols (FODMAP) diet and are linked to dysbiosis, which together lead to increased intestinal inflammation, barrier dysfunction, and visceral hypersensitivity (41).

Previous studies demonstrated that small molecule inhibitors of NHE3, including tenapanor, decrease intracellular pH, resulting in an increase in TEER and decreased intestinal permeability (28, 42). Intracellular acidification increases the rate of actin assembly in the cytoskeleton (43), which could mediate a decrease in paracellular permeability to luminal molecules through the expression and distribution of tight junction and adherens junction proteins, thereby restoring barrier function (42, 44). In IBS, such activity could counteract barrier dysfunction caused by dysbiosis, stress, or inflammation (41, 45). Here, tenapanor treatment in rats counteracted stress-induced VMR responses to CRD. Furthermore, while the inflammatory cytokines TNF-α and IL-6 increased epithelial cell permeability as measured by decreased TEER and increased permeability to the macromolecule FITC-dextran, treatment with tenapanor counteracted this cytokine-induced cell permeability and restored TEER to that of healthy epithelial cells. However, tenapanor did not affect the permeability of healthy epithelial cells (Fig. 7*E*), and prior research found that tenapanor did not reduce paracellular transport of mannitol in healthy rats (28). Thus, tenapanor may reduce visceral hypersensitivity and abdominal pain by limiting pathological paracellular absorption of molecules from the intestinal lumen and downstream enhanced immune responsiveness that can stimulate receptors such as TRPV1 (28).

We demonstrate that the beneficial effect of tenapanor on constipation in patients with IBS-C is due to increased luminal water retention, which facilitates GI transit. The beneficial effect of tenapanor on abdominal pain associated with IBS is likely mediated by a reduction of colonic paracellular permeability to luminal macromolecules and antigens, resulting in a restoration of normal TRPV1 signaling in hypersensitive DRG neurons. Future work is needed to determine how tenapanor treatment strengthens the intestinal barrier, and to identify the junctional proteins and signaling pathways that mediate this regulation of paracellular permeability.

## DATA AVAILABILITY

Data will be made available upon reasonable request based on data availability, burden, and data privacy issues. Requests should be sent to medinfo@ardelyx.com.

## GRANTS

Funding for medical writing and editorial assistance was provided by Ardelyx, Inc. Funding for the *Phase 1* trial was provided by Ardelyx, Inc. Funding for the nonclinical studies was provided by Ardelyx, Inc.

## DISCLOSURES

A. J. King, J. Wang, M. Siegel, J. S. Caldwell, S. Edelstein, D. P. Rosenbaum, and K. Kozuka were employees of Ardelyx, Inc., at the time the study was completed. L. Chang has served on a scientific advisory board and received research funding from Ardelyx, Inc. P. J. Pasricha has a sponsored research agreement with Ardelyx, Inc. He is a consultant for Vanda, FoodMarble, RxHealth, GI Scientific, and a founder of P4Microbiome and Neurogastrx. Q. Li, L. Liu, and Y. Zhu have nothing to disclose.

## AUTHOR CONTRIBUTIONS

A.J.K., Q.L., P.J.P., J.W., M.S., J.S.C., and S.E. conceived and designed research; L.C., L.L., Y.Z., and J.W. performed experiments; A.J.K., Q.L., P.J.P., and J.W. analyzed data; A.J.K., L.C., and J.S.C. interpreted results of experiments; P.J.P. prepared figures; Q.L., P.J.P., and K.K. drafted manuscript; A.J.K., L.C., Q.L., L.L., Y.Z., P.J.P., J.W., M.S., J.S.C., S.E., D.P.R., and K.K. edited and revised manuscript; A.J.K., L.C., Q.L., L.L., Y.Z., P.J.P., J.W., M.S., J.S.C., S.E., D.P.R., and K.K. approved final version of manuscript.
